# LncRNA *SNORD3A* specifically sensitizes breast cancer cells to 5-FU by sponging miR-185-5p to enhance UMPS expression

**DOI:** 10.1038/s41419-020-2557-2

**Published:** 2020-05-07

**Authors:** Liyun Luo, Jianlei Zhang, Hailin Tang, Dongfeng Zhai, Danqing Huang, Li Ling, Xiaorong Wang, Ting Liu, Qiong Zhang, Zhijie Zhang, Zhimin He, Guopei Zheng

**Affiliations:** 1 Affiliated Cancer Hospital & Institute of Guangzhou Medical University; Guangzhou Municipal and Guangdong Provincial Key Laboratory of Protein Modification and Degradation; The State Key Laboratory of Respiratory; Guangzhou Key Laboratory of “Translational Medicine on Malignant Tumor Treatment”, Hengzhigang Road 78#, 510095 Guangzhou, Guangdong China; 2 Department of Breast Oncology, State Key Laboratory of Oncology in South China, Sun Yat-sen University Cancer Center, Collaborative Innovation Center for Cancer Medicine, Dongfeng Road 651 E, 510060 Guangzhou, Guangdong China

**Keywords:** Breast cancer, Chemotherapy

## Abstract

Breast cancer is the most common cancer type in women. Long non-coding RNAs (lncRNAs) have been reported as potential new diagnostic markers, prognostic factors, and therapeutic targets in cancer. However, the specific roles and mechanisms of lncRNAs in breast cancer remain to be elucidated. Here we demonstrated the downregulation of lncRNA *SNORD3A* in breast cancer cells and tissues and verified its non-protein-coding property. *SNORD3A* overexpression had no effect on cell proliferation but specifically sensitized breast cancer cells to 5-fluorouracil (5-FU) in vitro and in vivo. Mechanistically, *SNORD3A* exerts its effect via enhancing uridine monophosphate synthetase (UMPS) protein expression. *SNORD3A* acts as a competing endogenous RNA for miR-185-5p, leading to UMPS protein upregulation. miR-185-5p overexpression disrupted the effect of *SNORD3A* on chemosensitization to 5-FU in vitro and in vivo. Moreover, Meis1 overexpression transcriptionally promotes *SNORD3A* expression, and Meis1 is downregulated in breast cancer cells and tissues. In breast cancer tissues, *SNORD3A* level positively correlates with Meis1 and UMPS protein levels, whereas miR-185-5p level negatively correlates with UMPS protein level. High *SNORD3A* transcript and Meis1 and UMPS protein levels predicts a better outcome, but high miR-185-5p level predicts a worse outcome in breast cancer patients receiving 5-FU-based chemotherapy. Our findings indicate that Meis1-regulated *SNORD3A* specifically sensitizes breast cancer cells to 5-FU via enhancing UMPS expression. The *SNORD3A*–UMPS axis may serve as a potential biomarker and therapeutic target to improve the efficacy of 5-FU-based chemotherapy for breast cancer patients.

## Introduction

Breast cancer is the most common cancer type in women, accounting for approximately 25% of new cancer cases^1^. Despite great advances in diagnosis and treatment strategies over the past decades, approximately 15% of the cancer-related deaths in women are due to breast cancer^2^. Breast cancer is a heterogeneous cancer type that exhibits a variety of histopathological characteristics and genetic alterations, with varying clinical outcomes. Based on intrinsic gene expression profiling, breast cancer can be divided into five major subtypes: luminal A, luminal B, HER2-positive breast cancer, triple-negative breast cancer (TN-BC), and normal breast-like subtype. TN-BC includes the basal-like and claudin-low subtypes^3–5^. The therapeutic strategy depends on the subtype and can include endocrine therapy, anti-HER2 targeting therapy, and chemotherapy. However, chemotherapy resistance remains a major obstacle for successful cancer treatment of all breast cancer types, resulting in recurrence, metastasis, and poor outcome^6^. Therefore, identifying strategies to overcome chemoresistance and increase chemosensitivity in breast cancer is critical to improve treatment efficacy.

Emerging evidence has revealed long non-coding RNAs (lncRNAs) as major regulators in both physiological and pathophysiological processes. LncRNAs are endogenous transcripts that are >200 nucleotides (nt) and lack protein-coding potential^7^. LncRNAs have been reported to be involved in a variety of biological activities, including proliferation, differentiation, migration, invasion, cell cycle, stem cell pluripotency, and lineage differentiation as well as cancer development and progression^8–10^. LncRNAs exert their roles at multiple mechanistic levels, such as regulating gene transcription, mRNA stability and translation, protein abundance and location, and chromatin and protein conformation^11,12^. Recent studies have revealed lncRNAs deregulation in a variety of cancer types and showed that lncRNAs can exhibit oncogenic function, cancer-suppressor function, or both^13–16^. Several lncRNAs have been shown to be involved in breast cancer. LncRNA NKILA binds nuclear factor (NF)-kB/inhibitor of kB (IkB) and masks the phosphorylation motif of IkB to inhibit IkB kinase-induced IkB phosphorylation and NF-kB activation, thus preventing breast cancer metastasis^17^. We previously reported that the lncRNA LINC01638 was highly expressed in the TN-BC subtype and LINC01638 activates MTDH-Twist1 signaling by preventing SPOP-mediated c-Myc degradation to maintain the epithelial–mesenchymal transition and cancer stem cell-like state of TN-BC cells^12^. However, the specific roles and mechanisms of lncRNAs in breast cancer are still not fully understood.

In this study, we investigated the expression and potential role of the lncRNA *SNORD3A* in breast cancer. We confirmed the downregulation of *SNORD3A* in breast cancer cells and tissues and demonstrated a novel mechanism by which *SNORD3A* regulates chemosensitivity to 5-fluorouracil (5-FU) in breast cancer.

## Materials and methods

### Cell culture, transfection and tissue samples

MCF10A, MCF-7, MDA-MB-231, T47D, SKBR3, ZR7530, BT549, HCC1937, BT474, and HEK293T cell lines were obtained from ATCC (Rockville, MD, USA) and cultured under standard conditions in media containing 10% fetal bovine serum (Gibco, Carlsbad, CA, USA).

To establish stable transfectants, cell lines were transfected with pReceiver-Lv201 lentiviral vectors containing *SNORD3A* or miR-185-5p, EX-T1651-Lv217 lentiviral vector containing *Meis1*, and psi-LVRU6GP vector with *UMPS* short hairpin RNAs (target sequence for sh-1#: 5’- CCAAUCAAAUUCCAAUGCU-3’, sh-2#: 5’-GAGUUGAUAACUCUGGCAA-3’) using Lipofectamine 3000 (Invitrogen, Carlsbad, CA, USA) following the manufacturer’s instructions.

To inhibit miR-185-5p function, cells were transfected with miR-185-5p inhibitor (miRCURY LNA™ microRNA inhibitor for miR-185-5p; Exiqon, Vedbaek, Denmark).

Frozen fresh and paraffin-embedded breast cancer and non-cancerous tissues were collected from patients at the Affiliated Cancer Hospital of Guangzhou Medical University. All samples were collected with informed consent from patients, and all procedures were performed after the internal review and approval of the Ethics Committees of Guangzhou Medical University and the Affiliated Cancer Hospital.

### RNA immunoprecipitation (RIP) assay

HEK293T cells were co-transfected with various cloned MS2bs vectors (MS2bs, MS2bs-*SNORD3A*-WT or MS2bs-*SNORD3A*-Mut) and MS2bp-GFP overexpression vector (Addgene, Watertown, MA, USA). After 48 h, RIP was performed with the EZ-Magna RIP Kit (Millipore, Burlington, MA, USA) using anti-green fluorescent protein (anti-GFP) according to the manufacturer’s instructions. After RNA extraction, miR-185-5p level was examined by quantitative reverse transcriptase polymerase chain reaction (qRT-PCR).

### Xenograft model in athymic mice

Female BALB/c athymic nude mice were obtained from Guangdong Medical Laboratory Animal Center, China. Cell lines were injected subcutaneously into the armpit of female BALB/c athymic nude mice to generate xenograft tumors (*n* = 5/group). Ten days after cancer cell implantation, mice were injected intraperitoneally with 5-FU (30 mg/kg) every 3 days for six cycles. At the experimental endpoint, animals were sacrificed, and then tumors were harvested and weighed. The animal studies were approved by the Institutional Animal Care and Use Committee (IACUC) of Guangzhou Medical University. Standard animal care and laboratory guidelines were conducted according to the IACUC protocol.

qRT-PCR, proliferation assay, colony-formation assay, chemosensitivity assay, western blot, luciferase reporter assay, chromatin immunoprecipitation (ChIP)-qPCR assay, in situ hybridization (ISH), immunohistochemistry (IHC) methods and primers are described in Supplemental Experimental Procedures.

### Statistical analysis

Data are presented as mean ± s.d. Student’s *t* test and χ^2^ test were used to compare the differences among different groups and correlation analysis. Survival curves were plotted using the Kaplan–Meier method and compared using log-rank test. Statistical analyses were performed using GraphPad Prism 6. *p* < 0.05 was considered statistically significant.

## Results

### LncRNA *SNORD3A* is downregulated in breast cancer

**Fig. 1:**
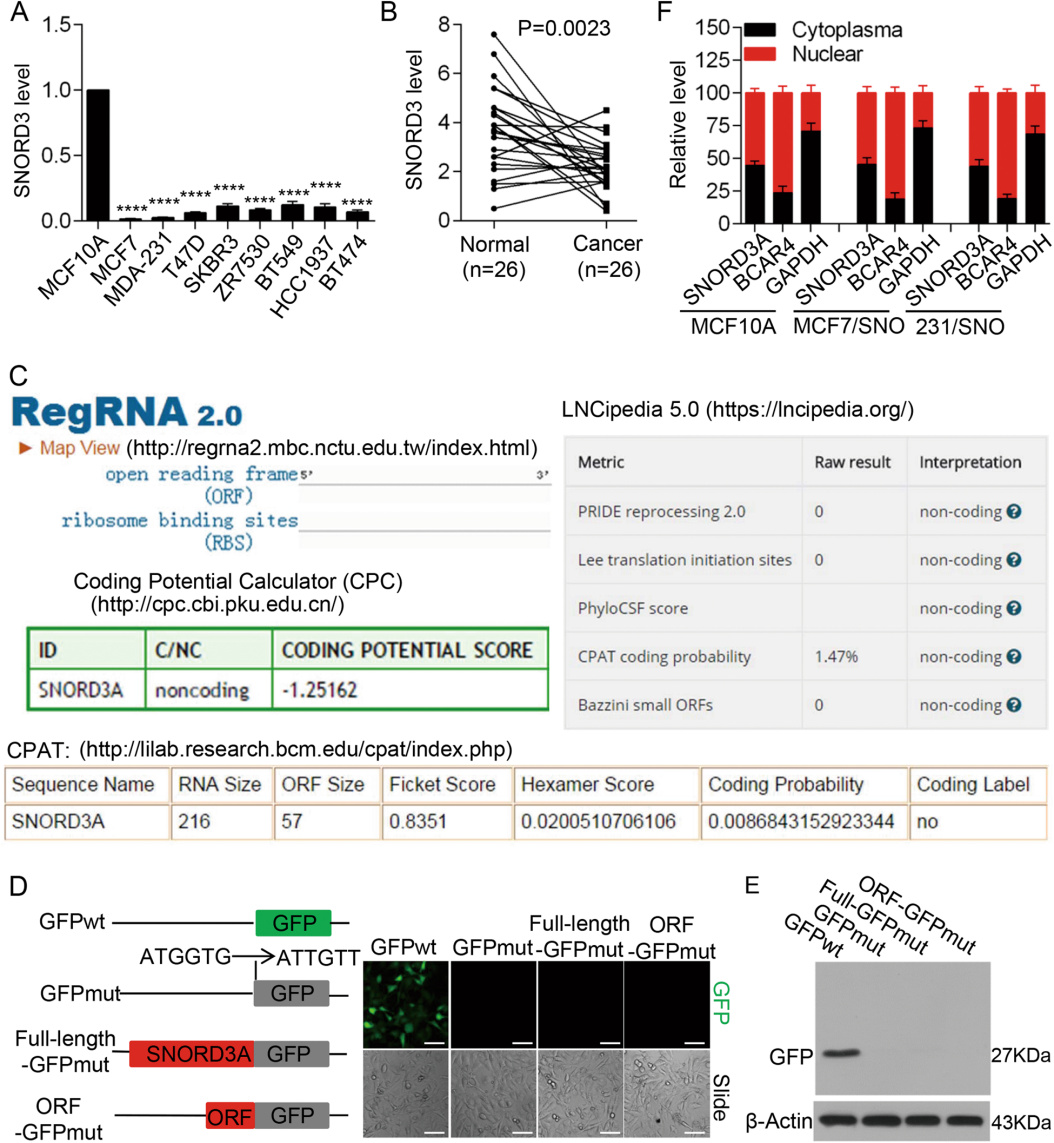
LncRNA *SNORD3A* is downregulated in breast cancer. **a** *SNORD3A* level was measured by qRT-PCR in a series of breast cancer cell lines. **b** *SNORD3A* level in paired breast cancer tissues and benign adjacent tissues was detected by qRT-PCR (*n* = 26). **c** Schematic of the coding potential and ribosome-binding sites of the *SNORD3A* transcript predicted by online analysis. **d**, **e** The full transcript of *SNORD3A* or potential ORF was cloned into the pEGFP-N1 vector with a mutation in the initiation codon of GFP and HEK293T cells were transfected as indicated. GFP fluorescence was observed (**d**) and GFP fusion protein levels were examined by western blot using anti-GFP antibody (**e**). Scale bar, 100 μm. **f** Fractionation of MCF10A, MDA-MB-231/SNORD3A, and MCF-7/SNORD3A cells followed by qRT-PCR. BCAR4 served as a positive control for nuclear fractions, and GAPDH functioned as a control for cytoplasmic fractions. Student’s *t* test, mean ± s.d. ****p < 0.0001.

We first examined the expression level of the annotated potential lncRNA *SNORD3A* in a series of breast cancer cell lines and the normal mammary epithelial cell line MCF10A. The results demonstrated that *SNORD3A* expression was downregulated in breast cancer cells compared with MCF10A cells (Fig. 1a). We then examined the expression pattern of *SNORD3A* in paired breast cancer tissues and adjacent non-cancerous tissues. *SNORD3A* expression was also downregulated in breast cancer tissues compared with levels in paired non-cancerous mammary tissues (Fig. 1b).

*SNORD3A* is a small nucleolar RNA located on human chromosome 17. *SNORD3A* is composed of three exons, with a transcript length of 699 nt. We analyzed the *SNORD3A* transcript using the online software (RegRNA 2.0, LNCipedia 5.0, Coding Potential Calculator), which predicted no protein-coding potentiality of *SNORD3A* (Fig. 1c). CPAT online software analysis also predicted no protein-coding ability but predicted an open reading frame (ORF) in the *SNORD3A* transcript (Fig. 1c).

To determine whether the *SNORD3A* ORF has coding potentiality, we constructed a series of vectors with a mutation in the initiation codon of GFP (the start codon ATGGTG was mutated to ATTGTT) that was fused downstream of the full *SNORD3A* transcript or potential ORF (Fig. 1d). While GFP expression was detected in HEK293T cells transfected with the wild-type GFP vector, no substantial expression of GFP was observed in HEK293T cells transfected with full-length-GFPmut or ORF-GFPmut construct (Fig. 1d). Western blot analysis using the anti-GFP antibody further confirmed that *SNORD3A* lacks protein-coding ability (Fig. 1e). We also examined the subcellular location of *SNORD3A* and found that *SNORD3A* resides in both the nucleus and the cytoplasm of breast cancer cells (Fig. 1f). These data indicated that *SNORD3A* as an lncRNA is downregulated in breast cancer cells and tissues.

### *SNORD3A* specifically enhances the chemosensitivity of breast cancer cells to 5-FU

To explore the biological function of *SNORD3A* in breast cancer, *SNORD3A* was stably overexpressed in MCF-7 and MDA-MB-231 cells (Fig. S1a). Cell proliferation assays showed that *SNORD3A* overexpression had no significant effect on proliferation (Fig. S1b). Colony-formation assay also showed that *SNORD3A* overexpression had no significant effect on colony-formation capacity of breast cancer cells (Fig. S1c).

**Fig. 2:**
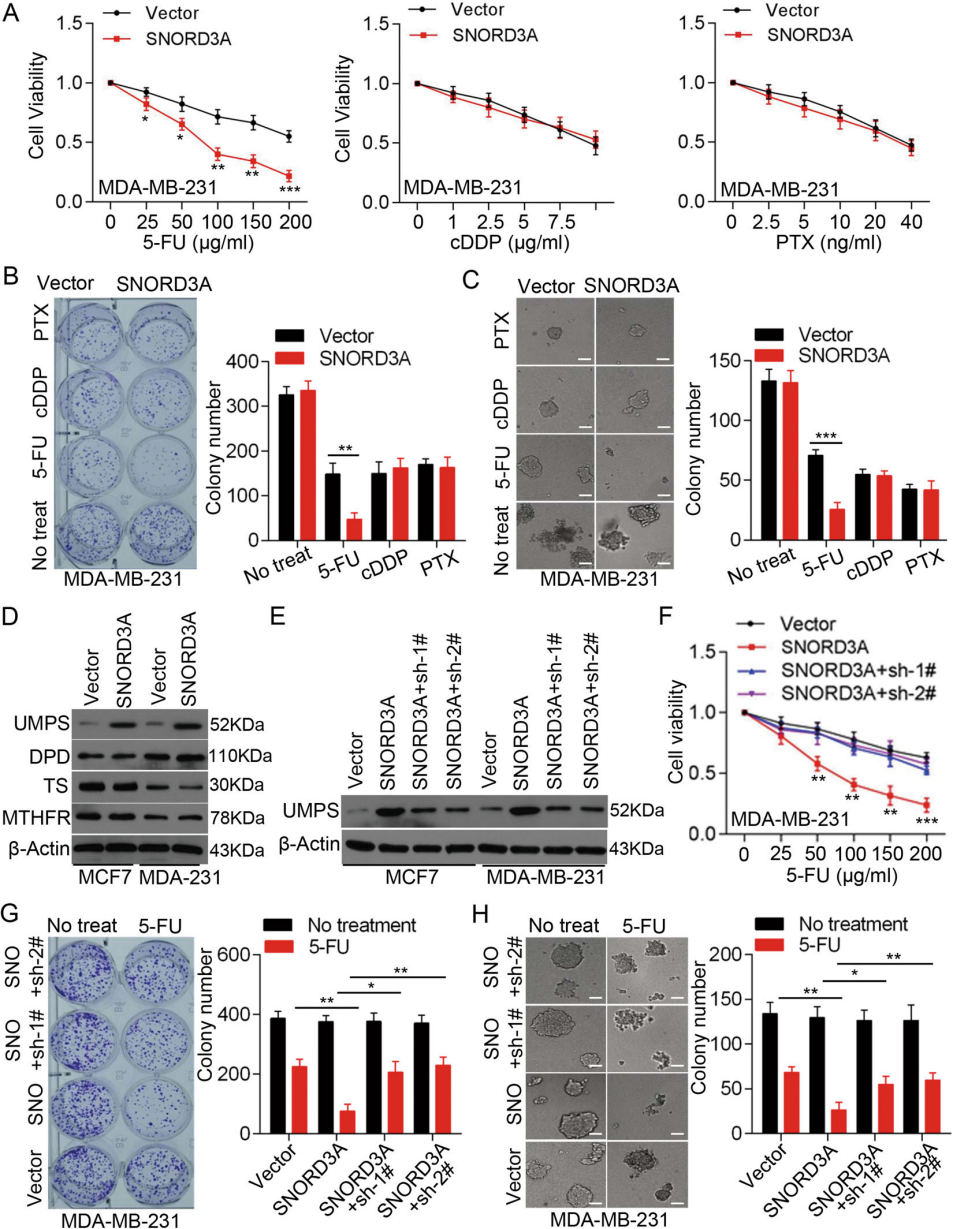
*SNORD3A* specifically enhances the chemosensitivity of breast cancer cells to 5-FU. **a** The effects of *SNORD3A* overexpression on the chemosensitivity of MDA-MB-231 cell to 5-FU, cisplatin (cDDP), and paclitaxel (PTX) were detected by MTS assay at 72 h post-treatment. **b**, **c** Colony-formation assay (**b**) and soft agar colony-formation assay (**c**) were performed to evaluate the colony growth of MDA-MB-231 cells under treatment with 5-FU (100 μg/ml), cDDP (5 μg/ml), or PTX (10 ng/ml). Scale bar, 100 μm. **d** Western blot showing UMPS, DPD, TS, and MTHFR protein levels in MCF-7 and MDA-MB-231 cells with or without *SNORD3A* overexpression. **e** UMPS protein levels in MCF-7 and MDA-MB-231 cells with *SNORD3A* overexpression and UMPS knockdown were examined by western blot. **f** Knockdown of UMPS abrogated the effects of *SNORD3A* overexpression on the chemosensitivity of MDA-MB-231 cells to 5-FU by MTS assay at 72 h post-treatment. **g**, **h** Colony growth of MDA-MB-231 cells with simultaneous expression interference of *SNORD3A* and UMPS were detected by colony-formation assay (**g**) and soft agar colony-formation assay (**h**). Scale bar, 100 μm. Student’s *t* test, mean ± s.d. *p < 0.05, **p < 0.01, ***p < 0.001.

We then examined whether *SNORD3A* was involved in the chemosensitivity of breast cancer cells. *SNORD3A*-overexpressing MCF-7 and MDA-MB-231 cells and controls were treated with 5-FU, cisplatin (cDDP), or paclitaxel (PTX) at different concentrations. We found that *SNORD3A* overexpression specifically enhanced the chemosensitivity of breast cancer cells to 5-FU but not to cDDP and PTX (Fig. 2a and Fig. S2a). Both plate colony-formation and soft agar colony-formation assays also confirmed that *SNORD3A* overexpression specifically promoted the chemosensitivity of breast cancer cells to 5-FU (Fig. 2b, c and Fig. S2b, c).

The 5-FU antimetabolite drug has been widely used for the treatment of different types of cancer. To investigate whether *SNORD3A* enhanced the chemosensitivity of breast cancer cells to 5-FU via modulating a metabolic pathway for 5-FU, we examined the expression of 5-FU metabolic-related genes, including uridine monophosphate synthetase (UMPS), thymidylate synthase (TS), dihydropyrimidine dehydrogenase (DPD), and methylene tetrahydrofolate reductase (MTHFR). *SNORD3A* overexpression had no effect on the expression of TS, DPD, and MTHFR in MCF7 and MDA-MB-231 cells, whereas *SNORD3A* overexpression markedly increased the UMPS protein level but not the mRNA level (Fig. 2d and Fig. S2d). To determine whether *SNORD3A* enhancement of the sensitivity to 5-FU in breast cancer cells is dependent on UMPS, we stably knocked down UMPS in breast cancer cells with ectopic *SNORD3A* overexpression (Fig. 2e). Knockdown of UMPS significantly diminished the promoting effect of *SNORD3A* overexpression on 5-FU sensitivity (Fig. 2f–h and Fig. S3a–c). Together, these data suggest that *SNORD3A* may promote 5-FU sensitivity by activating UMPS expression in breast cancer cells.

### *SNORD3A* promotes UMPS expression via interacting with miR-185-5p

**Fig. 3:**
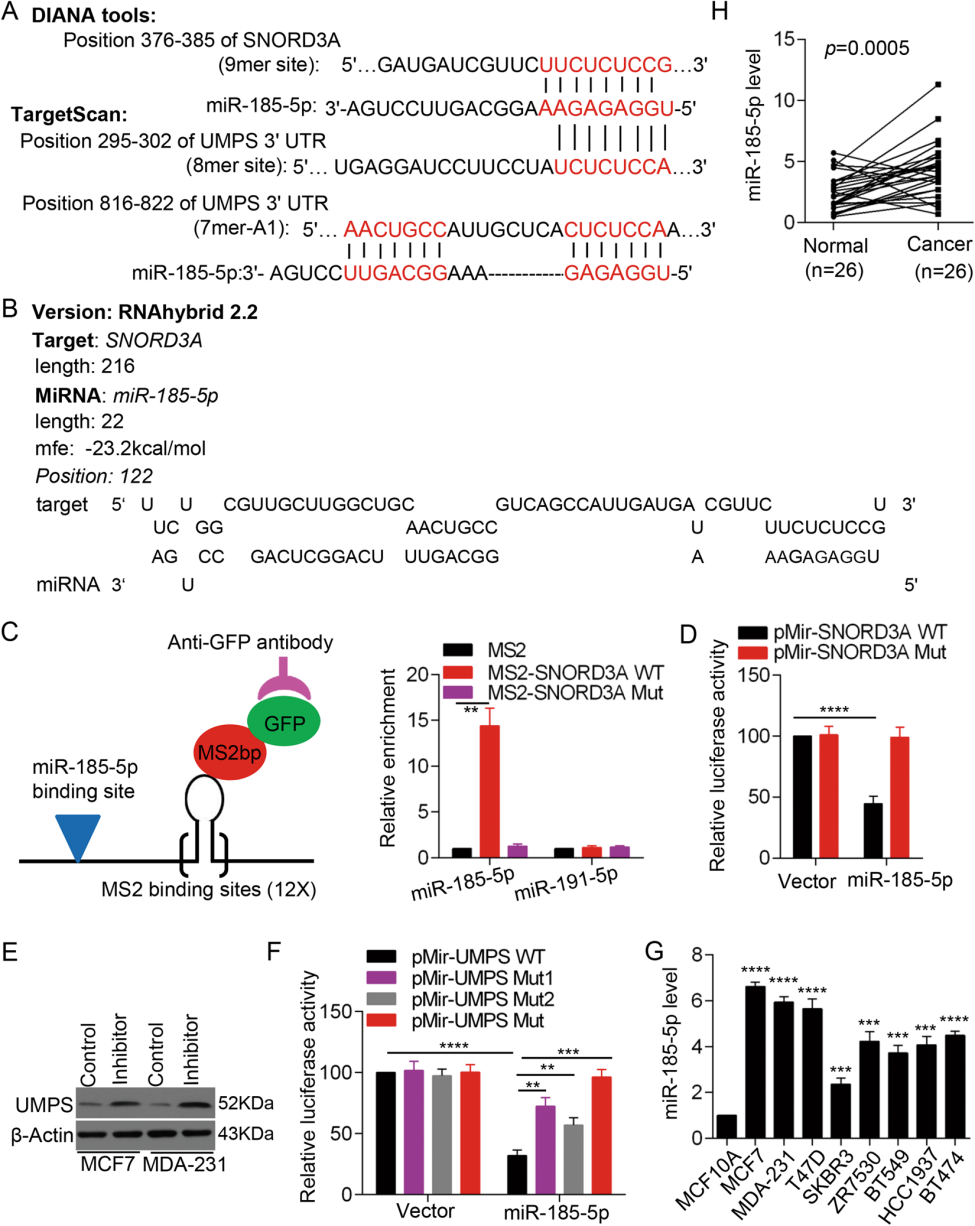
*SNORD3A* promotes UMPS expression via interacting with miR-185-5p. **a** Schematic illustration of the interaction between *SNORD3A* and miR-185-5p predicted with DIANA tools (http://carolina.imis.athena-innovation.gr/diana_tools/web/index.php) and between miR-185-5p and UMPS mRNA 3′-UTR predicted by TargetScan (http://www.targetscan.org/vert_72/). **b** The interaction between *SNORD3A* and miR-185-5p and the minimum free energy (mfe) were predicted with the RNAhybrid software (https://bibiserv.cebitec.uni-bielefeld.de/rnahybrid). **c** MS2-RIP assay followed by qRT-PCR to detect the association between *SNORD3A* and miR-185-5p. miR-191-5p served as a negative control. **d** Luciferase activity demonstrated that miR-185-5p targeted *SNORD3A*. **e** Western blot was performed to detect UMPS protein levels in MCF-7 and MDA-MB-231 cells after transfection with miR-185-5p inhibitor. **f** Luciferase activity indicated that miR-185-5p targeted UMPS. **g** miR-185-5p expression levels in human breast cancer cell lines were detected by qRT-PCR. **h** miR-185-5p levels in breast cancer tumor tissues and paired non-tumorous mammary tissues were examined by qRT-PCR (*n* = 26). Student’s *t* test, mean ± s.d. **p < 0.01, ***p < 0.001, ****p < 0.0001.

Our results demonstrated that *SNORD3A* overexpression increased UMPS protein level but did not alter UMPS mRNA level in breast cancer cells. We next investigated the mechanism by which *SNORD3A* regulates UMPS. Recent studies showed that lncRNAs can function as competing endogenous RNAs (ceRNAs) by acting as molecular sponges for microRNAs (miRNAs), thus regulating gene expression at the posttranscriptional level. We used the online DIANA tool (http://diana.imis.athena-innovation.gr) and TargetScan (http://www.targetscan.org) to screen potential miRNAs that may bind with the *SNORD3A* transcript and the 3’-untranslated region (UTR) of UMPS mRNA. We found that miR-185-5p may bind with *SNORD3A* transcript and UMPS mRNA 3’-UTR (Fig. 3a). The interaction between *SNORD3A* and miR-185-5p was further predicted, and the minimum free energy at the binding site was calculated using RNAhybrid (Fig. 3b).

To determine whether *SNORD3A* interacts with miR-185-5p via the predicted binding sites, wild-type *SNORD3A* or *SNORD3A* with deletion mutations in the miR-185-5p targeting site were cloned into the MS2b plasmid to transcribe RNA combined with MS2-binding sequences and then co-transfected with the MS2bp-GFP expression plasmid and miR-185-5p-expressing construct into HEK293T cells. We then performed RIP assays to pull-down miRNAs associated with *SNORD3A* via GFP antibody, followed by qRT-PCR. The results showed that miR-185-5p associated with *SNORD3A* via the targeting site (Fig. 3c). In contrast, miR-191-5p, a negative control, did not associate with *SNORD3A*. To further confirm the interaction of *SNORD3A* with miR-185-5p, we constructed luciferase reporters containing wild-type *SNORD3A* or *SNORD3A* with deletion mutation of the miR-185-5p targeting site. Luciferase constructs were co-transfected with miR-185-5p plasmid into HEK293T cells. We found that miR-185-5p reduced the reporter activity of the construct with wild-type *SNORD3A* (Fig. 3d).

We next examined whether UMPS is a direct target of miR-185-5p. miR-185-5p inhibitor upregulated UMPS protein levels in MCF-7 and MDA-MB-231 cells (Fig. 3e). We also constructed luciferase reporters containing the wild-type 3’-UTR UMPS mRNA or construct with mutated miR-185-5p targeting sites. miR-185-5p reduced the reporter activity of the construct with the wild-type 3’-UTR of UMPS mRNA (Fig. 3f), suggesting that miR-185-5p negatively regulates UMPS mRNA.

We further found that miR-185-5p expression was upregulated in breast cancer cell lines compared with the normal mammary epithelial cell line MCF10A (Fig. 3g). We also determined the expression of miR-185-5p in the cohort of the paired breast cancer and benign adjacent tissues and found that miR-185-5p levels were higher in breast cancer tissues than that in the paired non-cancerous mammary tissues (Fig. 3h). Collectively, these data suggested that miR-185-5p is highly expressed in breast cancer and that *SNORD3A* acts as a sponge to miR-185-5p to promote UMPS expression in breast cancer cells.

### miR-185-5p is involved in *SNORD3A*-mediated sensitization to 5-FU in breast cancer cells

**Fig. 4:**
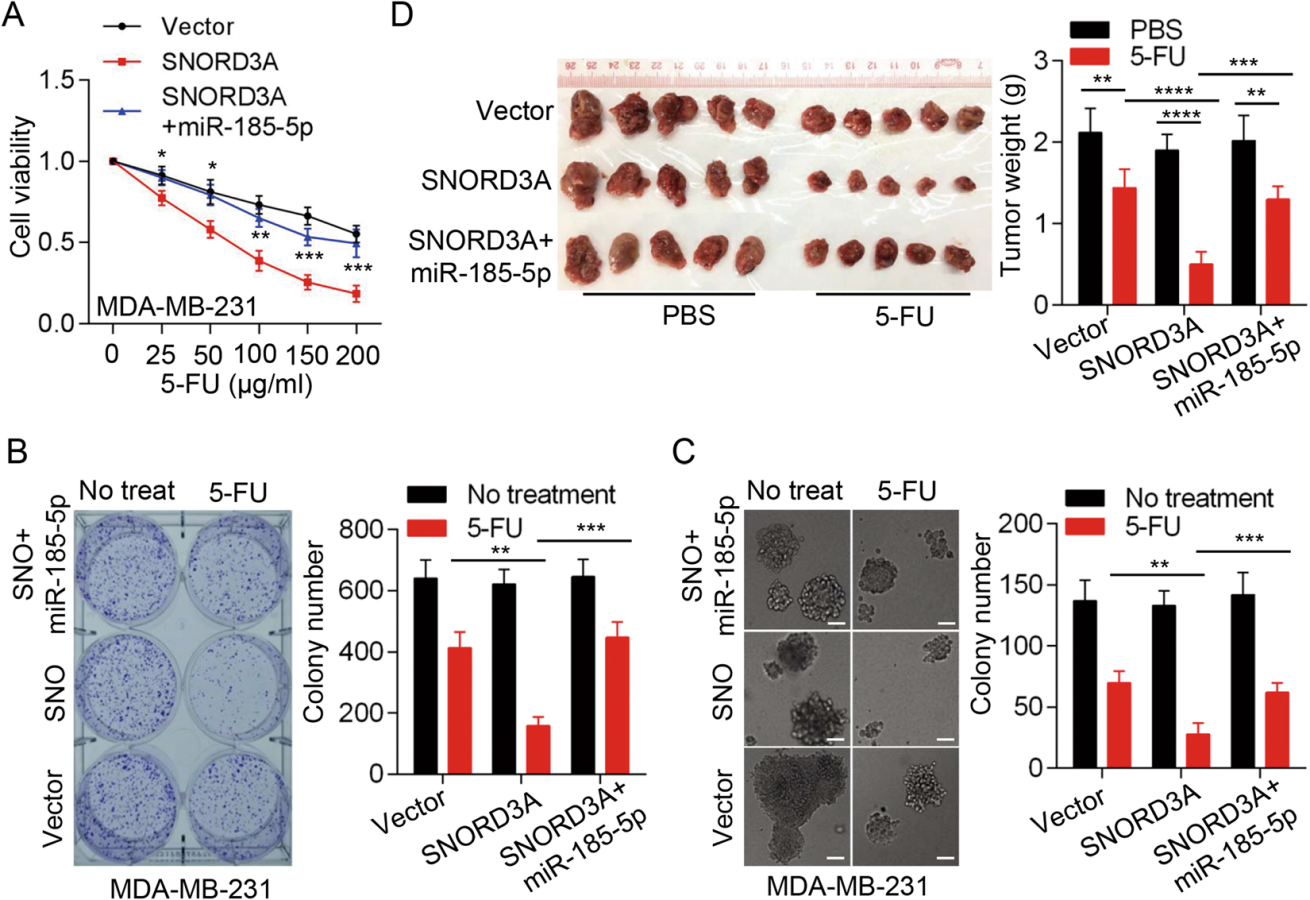
miR-185-5p is involved in *SNORD3A*-mediated chemosensitization to 5-FU in breast cancer cells. **a** Ectopic expression of miR-185-5p diminished the effects of *SNORD3A* overexpression on the chemosensitization of MDA-MB-231 cells to 5-FU by MTS assay at 72 h post-treatment. **b**, **c** Colony numbers of MDA-MB-231 cells with simultaneous expression interference of *SNORD3A* and miR-185-5p were detected by colony-formation assay (**b**) and soft agar colony-formation assay (**c**). Scale bar, 100 μm. **d** *SNORD3A* overexpression increased the chemosensitivity of MDA-MB-231-derived tumors to 5-FU, but co-expressed miR-185-5p abrogated the effect of *SNORD3A*. *n* = 5/group. Student’s *t* test, mean ± s.d. *p < 0.05, **p < 0.01, ***p < 0.001, ****p < 0.0001.

Given that *SNORD3A* enhanced chemosensitivity to 5-FU depending on UMPS and that *SNORD3A* acts as miR-185-5p sponge to promote UMPS expression in breast cancer cells, we examined the involvement of miR-185-5p in *SNORD3A*-mediated chemosensitization to 5-FU. miR-185-5p was stably overexpressed in MDA-MB-231 and MCF-7 cells with *SNORD3A* overexpression. MTS assays demonstrated that miR-185-5p overexpression diminished *SNORD3A*-induced sensitization to 5-FU (Fig. 4a and Fig. S4a). Moreover, both plate colony-formation and soft agar colony-formation assays confirmed that miR-185-5p overexpression reversed *SNORD3A*-induced sensitization to 5-FU (Fig. 4b, c and Fig. S4b, c).

We further evaluated the biological role of *SNORD3A* and miR-185-5p in vivo. We subcutaneously injected MDA-MB-231 cells with *SNORD3A* overexpression combined with or without miR-185-5p overexpression or control MDA-MB-231 cells into nude mice. Mice were then treated with 5-FU as described in “Methods.” *SNORD3A* overexpression significantly enhanced chemosensitivity to 5-FU compared with control mice treated with 5-FU. The volume and weight of tumors derived from *SNORD3A*-overexpressing cells were significantly decreased compared with those of control xenografts in response to 5-FU (Fig. 4d). However, miR-185-5p overexpression disrupted the effect of *SNORD3A* overexpression on chemosensitivity to 5-FU (Fig. 4d). Together these in vitro and in vivo results indicated that miR-185-5p is involved in *SNORD3A*-mediated sensitization to 5-FU in breast cancer cells.

### *SNORD3A* expression is regulated by Meis1 in breast cancer cells

**Fig. 5:**
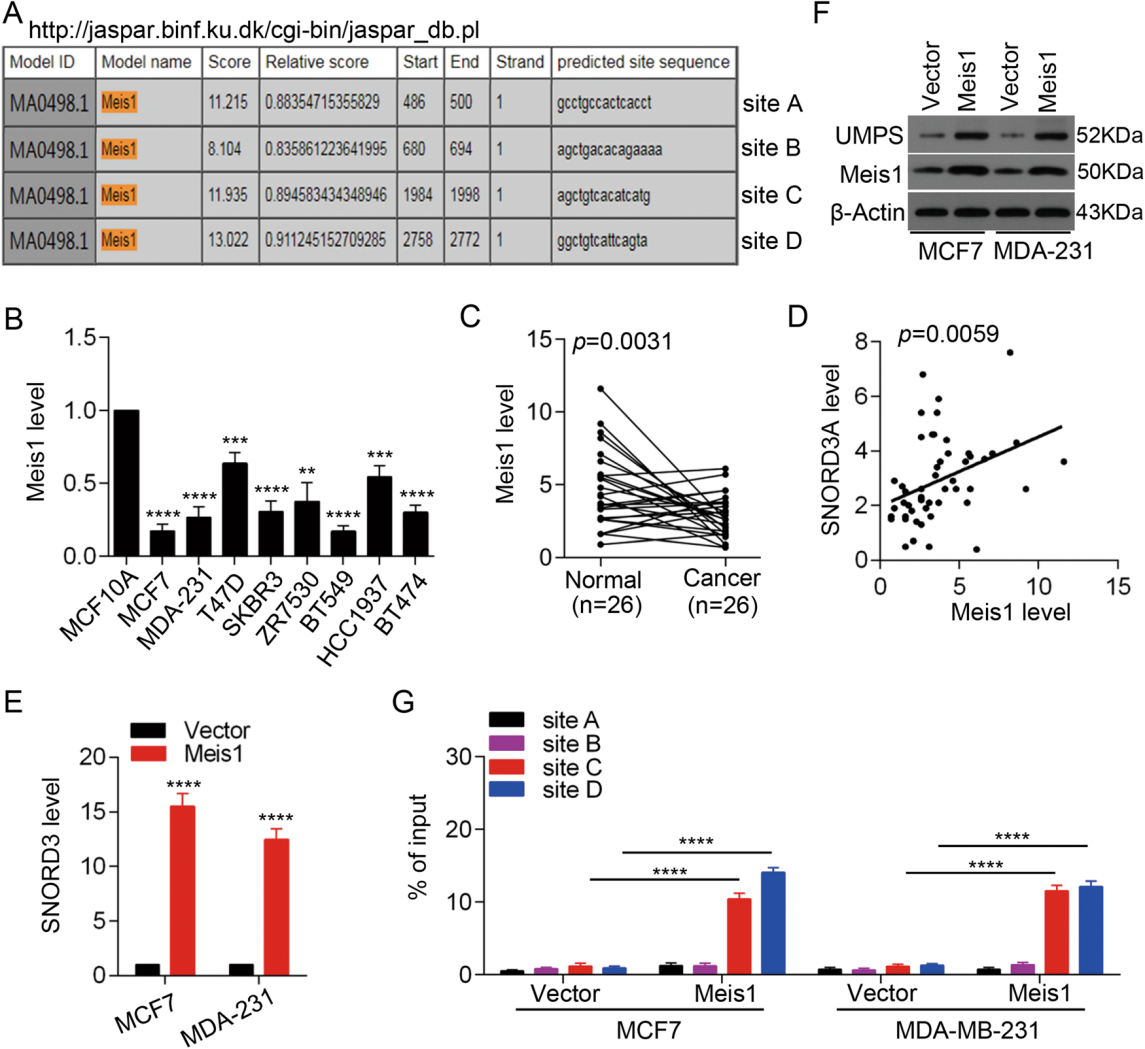
*SNORD3A* expression is regulated by Meis1 in breast cancer cells. **a** Schematic illustration of the Meis1-binding sites in the potential *SNORD3A* promoter predicted by JASPAR database (http://jaspar.binf.ku.dk). **b**, **c** qRT-PCR was performed to detect the expression levels of Meis1 in breast cancer cell lines (**b**) and in paired breast cancer tissues and normal mammary tissues (*n* = 26) (**c**). **d** The correlation between Meis1 and *SNORD3A* expression in breast cancer tissues was analyzed. **e**, **f** Meis1 overexpression resulted in elevated mRNA level of *SNORD3A* (**e**) and protein level of UMPS in MCF-7 and MDA-MB-231 cells (**f**). **g** Enrichment of Meis1 protein on the *SNORD3A* promoter was determined by ChIP-qPCR assay. Student’s *t* test, mean ± s.d. **p < 0.001, ***p < 0.001, ****p < 0.0001.

Given that *SNORD3A* expression was downregulated in breast cancer cells and tissues, we next explored the mechanisms underlying *SNORD3A* downregulation. To identify the transcription factors that regulate *SNORD3A* transcription, we used the JASPAR database (http://jaspar.binf.ku.dk) to predict potential transcription factor-binding sites in the 2-kb region upstream of the transcription start site in the *SNORD3A* gene. The analysis identified four potential Meis1-binding sites in the *SNORD3A* promoter (Fig. 5a). We further found that Meis1 expression was downregulated in breast cancer cells compared with the normal mammary cell line MCF10A (Fig. 5b). Meis1 level was also decreased in breast cancer tissues from the cohort compared with non-cancerous tissues (Fig. 5c). Correlation analysis indicated that Meis1 level was positively correlated with *SNORD3A* level in breast cancer tissues (Fig. 5d). To investigate the potential role of Meis1 in regulating *SNORD3A* expression, Meis1 was overexpressed in MCF-7 and MDA-MB-231 cell lines. Meis1 overexpression increased *SNORD3A* expression (Fig. 5e) as well as UMPS, the downstream target of *SNORD3A* (Fig. 5f). We next performed ChIP assays to confirm Meis1 occupancy on the *SNORD3A* promoter and found that Meis1 overexpression resulted in enrichment of Meis1 at the predicted Site C and Site D in the *SNORD3A* promoter in MCF-7 and MDA-MB-231 cells (Fig. 5g). Together these results suggested that Meis1 transcriptionally regulates *SNORD3A* expression in breast cancer cells.

### Clinical significance of *SNORD3A*-related signaling in breast cancer patients

**Fig. 6:**
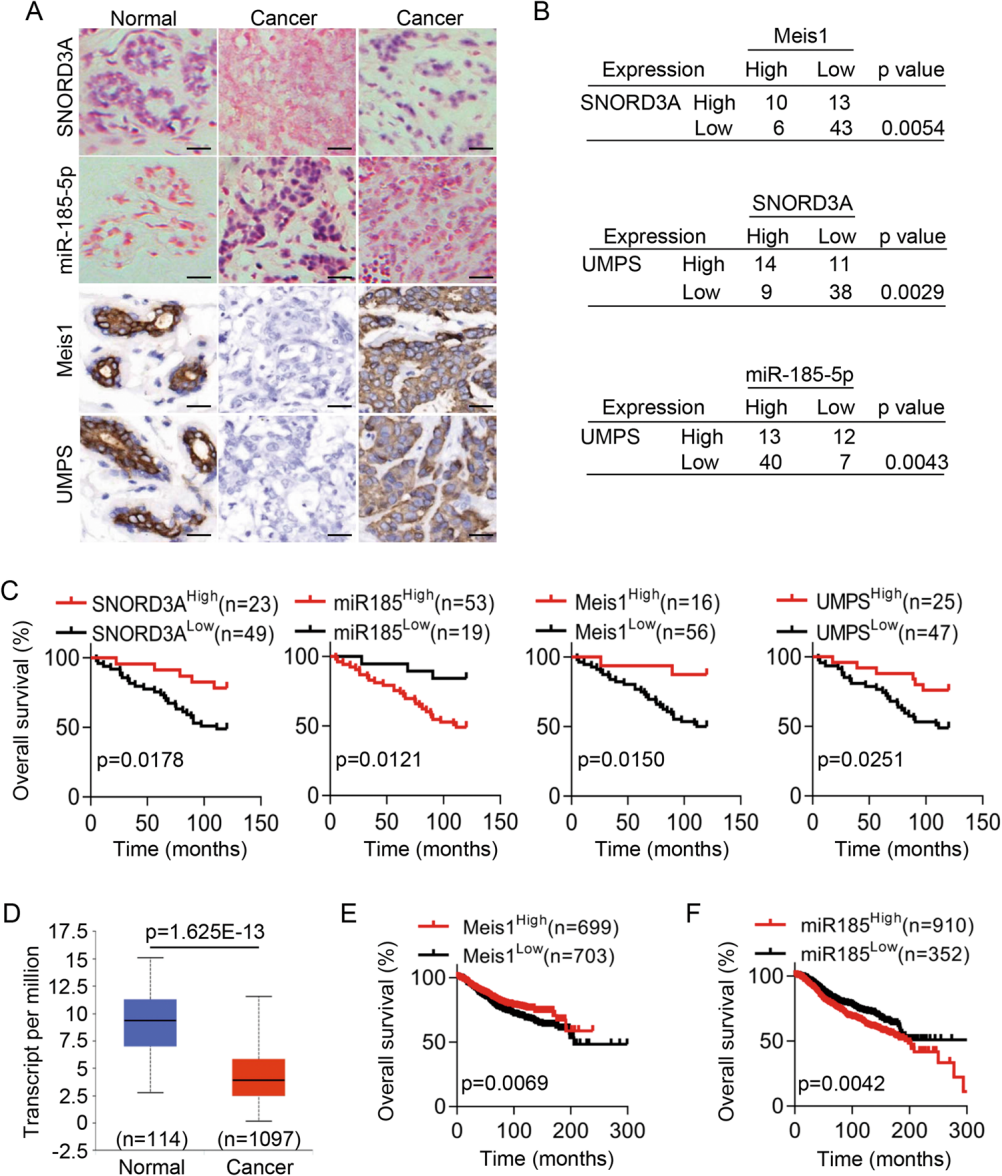
Clinical significance of *SNORD3A*-related signaling in breast cancer patients. **a** *SNORD3A* transcript and miR-185-5p levels in breast cancer tissue samples were detected by in situ hybridization (ISH). Meis1 and UMPS protein levels in breast cancer tissue samples were detected by immunohistochemical staining (IHC) (*n* = 72). Scale bar, 50 μm. **b** Correlations between *SNORD3A* transcript level and Meis1 protein level, between UMPS protein level and *SNORD3A* transcript level, and between UMPS protein level and miR-185-5p level in breast cancer tissues were analyzed. **c** Kaplan–Meier analysis indicated a correlation between low *SNORD3A* transcript level, Meis1 protein level, or UMPS protein level and poor overall survival in breast cancer patients and a correlation between high miR-185-5p level and poor overall survival in breast cancer patients. **d** UALCAN (http://ualcan.path.uab.edu) showed that Meis1 expression was downregulated in breast cancer tissues. **e**, **f** Kaplan–Meier plots according to Meis1 (**e**) and miR-185-5p (**f**) expression were generated for breast cancer patient cohorts in the TCGA database. Log-rank *p* values are shown.

To further define the role of *SNORD3A* and verify its correlation with the newly identified upstream regulator and downstream effectors in breast cancer clinical samples, we first examined *SNORD3A* and miR-185-5p levels via ISH in breast cancer tissues (*N* = 72). *SNORD3A* transcript was expressed at low levels in approximately 68.06% of the specimens, while miR-185-5p transcript was highly expressed in approximately 73.61% of the specimens (Fig. 6a). We performed IHC staining of Meis1 and UMPS proteins in breast cancer tissues that were examined by ISH. As expected, Meis1 and UMPS protein were expressed at low levels in approximately 77.78% and 65.28% breast cancer specimens, respectively (Fig. 6a). Further analysis showed that Meis1 level was positively correlated with *SNORD3A* level. UMPS level was positively correlated with *SNORD3A* level but negatively correlated with miR-185-5p level (Fig. 6b). Importantly, high *SNORD3A* transcript and Meis1 and UMPS protein levels predicts a better outcome, but high miR-185-5p level predicts a worse outcome in breast cancer patients receiving 5-FU-based chemotherapy (Fig. 6c).

We then used online tools to analyze the expression pattern and prognosis in breast cancer. Online analysis using UALCAN (http://ualcan.path.uab.edu) based on 1097 breast cancer tissues and 114 non-tumor mammary tissues indicated that Meis1 was downregulated in breast cancer tissues (Fig. 6d). We used a registration-free online service to generate Kaplan–Meier plots and found that high Meis1 expression in breast cancer predicted a better outcome in breast cancer (Fig. 6e), while high miR-185-5p predicted a worse outcome (Fig. 6f). There were no available records for *SNORD3A* in the online database. Together these data demonstrated the clinical significance of *SNORD3A*-mediated signaling in breast cancer patients.

## Discussion

In this study, we for the first time delineated the expression pattern, critical role, and mechanisms of the lncRNA *SNORD3A* in breast cancer. Our findings provide several insights into the underlying mechanisms and potential strategy for enhancing 5-FU chemosensitivity in breast cancer: (1) *SNORD3A* as an lncRNA is downregulated in breast cancer (2) resulting from downregulation of the Meis1 transcription factor and (3) *SNORD3A* acts as a ceRNA of miR-185-5p (4) to upregulate UMPS protein expression and specifically enhance chemosensitivity to 5-FU.

Emerging evidence has revealed the aberrant expression of lncRNAs in cancer and their critical roles in cancer development, progression, and prognosis^18^. Some lncRNAs have been reported to regulate important biological processes in cancer, such as proliferation^19^, metabolism^20^, cancer stem cells^15^, and metastasis^21^. LncRNAs deregulation has also been shown to be involved in the chemotherapeutic response and chemoresistance that is correlated with eventual cancer mortality^22^. Here we demonstrated low expression of lncRNA *SNORD3A* in breast cancer cells and tissues. *SNORD3A* overexpression had no significant effects on breast cancer cell proliferation and growth but specifically enhanced the chemosensitivity to 5-FU, validated by in vitro and in vivo studies. Importantly, higher *SNORD3A* level was positively correlated with clinical outcome in breast cancer patients receiving 5-FU-based chemotherapy. 5-FU is a pyrimidine analog that disrupts nucleoside metabolism and a widely used chemotherapy agent for some cancer types including breast cancer and colon cancer^23^. 5-FU is converted into its active metabolite fluorouridine monophosphate (FUMP), either directly by the orotate phosphoribosyltransferase domain of UMPS combining with phosphoribosyl pyrophosphate or indirectly via the activity of fluorouridine. FUMP is further phosphorylated and converted to fluorouridine triphosphate (FUTP). 5-FU induces cytotoxic effects and cell death through the incorporation of its active metabolite FUTP into RNA^24^. Abnormal alteration in the expression of 5-FU pathway genes directly correlates with chemosensitivity to 5-FU^25^. Here we demonstrate that *SNORD3A* enhanced chemosensitization via inducing UMPS expression at the protein level and showed that UMPS knockdown impeded *SNORD3A*.

Given that *SNORD3A* increased UMPS expression at the protein level but not at the mRNA level, we next explored the molecular mechanism by which *SNORD3A* regulates UMPS expression. Some lncRNAs function as ceRNAs to regulate the functions of miRNAs through miRNA response elements^26^. The lncRNA H19 promotes 5-FU resistance in colorectal cancer (CRC) by sponging miR-194-5p and regulating SIRT1-mediated autophagy^27^. The lncRNA HOTAIR promotes gastric cancer progression by sponging miR-331-3p to upregulate HER2 expression^28^. Exosome-transmitted lncARSR functions as a sponge of miR-34/miR-449 to induce c-MET and AXL expression to mediate sunitinib resistance in renal cell carcinoma^29^. In this study, we found that *SNORD3A* shared miR-185-5p response elements with UMPS and facilitated UMPS expression by sponging miR-185-5p. miR-185-5p was highly expressed in breast cancer, which predicted a poor outcome in breast cancer patients. UMPS was experimentally validated to be a bona fide target of miR-185-5p. Ectopic miR-185-5p expression overcame the chemosensitization induced by *SNORD3A* in in vitro and in vivo models, indicating that *SNORD3A* acts as a ceRNA for miR-185-5p in breast cancer cells.

Recent studies reported the tumor-suppressive roles of miR-185-5p in cancer progression. miR-185 function is blocked by miR155HG, resulting in ANXA2 upregulation and glioblastoma growth and progression^30^. In CRC, miR-185 downregulation by TCF1/LEF1 contributed to DC-SIGN-induced cancer metastasis^31^. Interestingly, in non-small cell lung cancer, miR-185 inhibited cancer growth and invasion but increased chemosensitivity^32^. Here we showed that *SNORD3A* enhanced the sensitivity of breast cancer cells to 5-FU via sponging miR-185-5p and that miR-185-5p acts as a suppressive miRNA of UMPS to mediate the insensitivity to 5-FU. Thus miR-185-5p may exhibit different effects in cancer progression and the therapeutic response. The precise effects of miR-185-5p and detailed mechanisms in cancer should be further validated by independent cohorts and prospective studies.

We also explored preliminary the mechanism for *SNORD3A* downregulation in breast cancer. Bioinformatics analysis combined with experimental validation indicated that Meis1 was downregulated in breast cancer and positively regulated *SNORD3A* expression at the transcriptional level. Clinically, low Meis1 level predicts a poor prognosis in breast cancer patients. Meis1, a 3-amino acid loop extension class transcription factor, activates its target genes via interacting with Hox transcription factors^33^. Meis1 plays important roles in tumorigenesis, such as in the development of neuroblastomas^34^, ovarian carcinomas^35^, and leukemia^36^. Sebastian et al. reported that Meis1 enhances Syk signaling via downregulating miR-146a expression in Hoxa9-driven acute myeloid leukemia^37^. The detailed molecular mechanisms for Meis1 transcriptionally regulating SNORD3A will be investigated in our next studies.

In conclusion, our study delineates the expression pattern, role, and mechanism of the lncRNA *SNORD3A* in breast cancer. *SNORD3A* is downregulated in breast cancer resulting from Meis1 downregulation. *SNORD3A* enhances UMPS expression by sponging miR-185-5p to specifically promote chemosensitivity to 5-FU in breast cancer. Our findings provide insight into the *SNORD3A*–UMPS axis as a promising therapeutic target against breast cancer, with important translational implications for improving the efficacy of 5-FU for breast cancer patients. Further studies should be performed to develop precise strategies targeting *SNORD3A*–UMPS signaling.

## Supplementary information

Supplemental Information

Figure S1

Figure S2

Figure S3

Figure S4
