# Supplementary material for: LncRNA SNORD3A specifically sensitizes breast cancer cells to 5-FU by sponging miR-185-5p to enhance UMPS expression
Source: Cell Death Dis. 2020 May 7;11(5):329. doi: 10.1038/s41419-020-2557-2 (PMC7205983; doi:10.1038/s41419-020-2557-2)
Supplement: Supplementary file 1 — Supplemental Information [file 41419_2020_2557_MOESM1_ESM.docx]

Supplemental Information

LncRNA *SNORD3A* specifically sensitizes breast cancer cells to 5-FU by sponging miR-185-5p to enhance UMPS expression

Supplementary Data

Figur S1, related to Figure 2. *SNORD3A* specifically enhances the chemosensitivity of breast cancer cells to 5-FU.

(A) qRT-PCR was performed to detected the *SNORD3A* overexpression after stable cell lines were established. (B) The effects of *SNORD3A* expression on the proliferation potential of BC cells were determined by a cell number count. (C) The effects of *SNORD3A* overexpression on colony formation capacity of BC cells were measured by the plate colony formation assay. Student’s t-test, Mean±s.d. _****_ *p* < 0.0001.

Figure S2, related to Figure 2. *SNORD3A* specifically enhances the chemosensitivity of breast cancer cells to 5-FU.

(A) The effects of *SNORD3A* overexpression on chemosensitivity of MCF-7 cells to 5-FU, Cisplatin (cDDP) and Paclitaxel (PTX) were detected by MTS assay at 72 hr post-treatment. (B-C) The plate colony formation assay (B) and soft gar colony formation assay (C) were performed to evaluate the colony number of MCF-7 cells under treatment with 5-FU (100μg/ml), cDDP (5μg/ml) or PTX (10ng/ml). Scale bar, 100 μm. (D) The UMPS, DPD, TS and MTHFR transcript levels in MCF-7 and MDA-MB-231 cells with *SNORD3A* overexpression were detected by qRT-PCR. Student’s t-test, Mean±s.d. _*_ *p* < 0.05, _**_ *p* < 0.01, _***_ *p* < 0.001.

Figure S3, related to Figure 2. *SNORD3A* specifically enhances the chemosensitivity of breast cancer cells to 5-FU.

(A) Knockdown of UMPS abrogated the effects of *SNORD3A* overexpression on the chemosensitivity of MCF-7 cells to 5-FU by MTS assay at 72 hr post-treatment. (B-C) The colony number of MCF-7 cells with simultaneous expression interference of *SNORD3A* and UMPS were detected by the plate colony formation assay (B) and soft gar colony formation assay (C). Scale bar, 100 μm. Student’s t-test, Mean±s.d. _**_ *p* < 0.01, _***_ *p* < 0.001.

Figure S4, related to Figure 4. miR-185-5p is involved in *SNORD3A* mediated chemosensitization to 5-FU in breast cancer cells.

(A) Ectopic expression of miR-185-5p diminished the effects of *SNORD3A* overexpression on the chemosensitization of MCF-7 cells to 5-FU by MTS assay at 72 hr post-treatment. (B-C) The clones number of MCF-7 cells with simultaneous expression interference of *SNORD3A* and miR-185-5p were detected by the plate colony formation assay (B) and soft gar colony formation assay (C). Scale bar, 100 μm. Student’s t-test, Mean±s.d. _**_ *p* < 0.01, _***_ *p* < 0.001.

Supplemental Experimental Procedures

RNA extraction and quantitative real-time PCR analyses (qRT-PCR)

Total RNA was isolated from cells and frozen tissues using the TRIzol reagent (Invitrogen, USA), and 4 μg of total RNA was reverse-transcribed into cDNA using an RT-PCR reverse transcription kit (Thermo Fisher Scientific, USA) to detect relative mRNA. The cDNA was used for quantitative RT-PCR (qRT-PCR) using the SYBR Green I Kit (Thermo Fisher Scientific, USA) according to the manufacturer’s protocol. For the miRNAs, 2.5 μg of total RNA was reverse transcribed using All-in-One MiRNA Q-PCR Detection Kit (GeneCopoeiaTM, China) according to the manufacturer’s protocol. GAPDH was performed as an internal reference for cytoplasmic gene expression, while BCAR4 was performed as a positive control for gene expression in nucleus. The 2^−ΔΔCt^ method was used to calculate the relative expression of each gene relative to the amount of GAPDH, and each qRT-PCR was performed in triplicate and the mean values were calculated. The primers for qRT-PCR: *SNORD3A*: forward primer: 5'-CGGTGACGGCTCTTGGGTTT-3', reverse primer: 5'-CGGGAAACGGCGACAAAA-3'; UMPS: forward primer: 5'-TTGGTGACGGGTCTGTACGA-3', reverse primer: 5'-GAAGACGCGGTCGAGACAC-3'; Meis1: forward primer: 5'-GGGCATGGATGGAGTAGGC-3', reverse primer: 5'-GGGTACTGATGCGAGTGCAG-3'; GAPDH: forward primer: 5'-AATGGGCAGCCGTTAGGAAA-3', reverse primer: 5'-GCCCAATACGACCAAATCAGAG-3'.

Proliferation assay

Cell proliferation was monitored by cell number count assay, 1×10^3^ cells per well were seeded in triplicate into 96-well plates. After 24 hr, the numbers of cells were measured in triplicate every day for 6 days. The curves were then constructed by calculating the mean value of the cell number.

Plate colony formation assay

Cells were seeded into 6-well plates at 250, 500, 1000 cells/well in triplicates and incubated to allow colony formation for 8-12 days. Alternatively, cells were seeded in 12-well plate at 1,000 cells per well 5-FU (100 µg/ml), cDDP (5 µg/ml) or PTX (10 ng/ml) in 1 ml of media was added to the cells. Following 8-12 days of incubation. Then, the colonies were fixed with methanol and stained with crystal violet. Cell colonies were quantified in per well fields, and the mean value was calculated.

Soft agar colony formation assay

500 cells were suspended in medium containing 0.35% agar and 10% FBS with 5-FU (100 µg/ml) or cDDP (5 µg/ml), PTX (10 ng/ml) and layered on medium containing 0.7% agar and 10% FBS in 12-well plate. Colonies were following 3 weeks of incubation. Colonies from three replicate wells were quantified.

MTS assay

Cells were seeded at a density of 5000 cells per well in 96-well plates. After 24h incubation, and then treated with 5-FU, cDDP or PTX at different concentrations for 72 hr. MTS solution (20 µl) was added to each well. After incubation for 3 h at 37℃, absorbance value was measured at 490 nm and the relative cell viability was calculated.

Western blot

Total protein was lysed with RIPA Lysis Buffer (Thermo Scientific, Rockford, IL, USA) with Protease and Phosphatase inhibitors (Halt Protease and Phosphatase Inhibitor Cocktail, Thermo) for 10 min on ice. The lysates were centrifuged 12,000 g for 15 min. The protein concentration was measured by BCA Protein Assay Kit (Thermo, USA). 30 μg of protein was used for separation by 10% SDS-PAGE gels and transferred onto 0.22 μm PVDF membranes (Merck Millipore, Schwalbach, Germany). The membrane was blocked with 5% non-fat milk in TBS-Tween (TBS-T, 0.1 % Tween) at room temperature for 2 hr and incubated overnight at 4 °C with the primary antibodies. The membrane was incubated with horseradish peroxidase (HRP)-conjugated secondary antibodies (dilution 1:5000; Sigma-Aldrich) at room temperature for 2 hr. Finally, the membranes were washed and the immunoreactive bands were visualized using an ECL western blotting system (Thermo, USA). The following antibodies were used: a monoclonal mouse anti-β-actin antibody (#A5316, Sigma, USA), a monoclonal mouse anti-UMPS antibody (sc-398086, Santa Cruz Biotechnology, USA), a monoclonal rabbit anti-Meis1 antibody (#MA5-27191, Invitrogen, USA), a monoclonal rabbit anti-DPD antibody (#4654, CST, USA), a monoclonal rabbit anti-TS antibody (#9045, CST, USA), a monoclonal rabbit anti-MTHFR antibody (#25164, CST, USA).

Luciferase reporter assay

HEK293T cells were co-transfected with pMir-*SNORD3A*-WT or pMir-*SNORD3A*-Mut, pRL-TK Renilla luciferase vector and miR-185-5p plasmid. Similarly, pMir-UMPS-WT or pMir-UMPS-Mut was co-transfected with pRL-TK Renilla luciferase vector and miR-185-5p plasmid into HEK293T cells using Lipofectamine 3000 (Invitrogen, USA). 48hr after transfection, the relative luciferase activity was normalized to Renilla luciferase activity using the Dual-Luciferase Reporter Assay System (Promega, USA) on a BioTek Synergy 2.

ChIP-qPCR

EZ-ChIP™ Chromatin immunoprecipitation kit (Millipore, USA) was utilized to perform ChIP assays via the manufacturer's protocol. Briefly, 2×10^7^ cells were fixed with 1% formaldehyde for 10 min and neutralized by glycine for 5 min at room temperature, cells were then lysed for 10 min and sonicated for 10 times (20 seconds on, 10 seconds off) on ice. Supernatant was prepared for immunoprecipitation with 5 µg of anti-Meis1 antibody (Abcam, USA), polymerase II, and normal rabbit IgG, respectively. The complexes were incubated overnight at 4°C. Pre-blocked protein G magnetic beads were added to the solution for 1 hr with gentle inversions. Cross-links were then reversed to free DNA by the addition of 5M NaCl and incubation at 65°C for 4 h. RNA was digested with RNase A and proteins were digested with Proteinase K at 65°C for 4 hr, DNA was purified and qRT-PCR was used to verify the interaction between *SNORD3A* and Meis1. Primers for the *SNORD3A* promoter-with Meis1 binding sites: For site A: forward primer, 5'-TGATAGTTTATTGTTTTTGAGTTTT-3' and reverse primer, 5'- CAGATTGGGATAGTAGAATGTAGAG -3'. For site B: Forward primer, 5'-TCAAACAACACTTGCTTACCC-3' and reverse primer, 5'- ACTTTTAAACATTACTTGTGCCAC-3'. For site C: Forward primer, 5'-CAGGACTAACATTACTGCTGGGGA-3' and reverse primer, 5'-GAAAGTCTTATGTGTGTAGCCAGGG-3'. For site D: Forward primer, 5'-TCCCACTCCTTTTCCACAGTCTCC-3' and reverse primer, 5'-GTACCGGGCAGCATACTTTCTTGAG-3'. Primers for the human GAPDH gene: forward primer, 5′-AATGGGCAGCCGTTAGGAAA-3′ and reverse primer: 5′-GCCCAATACGACCAAATCAGAG-3'.

In situ hybridization (ISH)

ISH assay was performed to detect the expression of *SNORD3A* and miR-185-5p in breast cancer tissues according to manufacturer’s procedures (Exiqon, Vedbaek, Denmark). For paraffin-embedded tissue, after deparaffinization and rehydration, the samples were treated with proteinase K, and then incubated in prehybridization buffer, probed with *SNORD3A* and miR-185-5p probe tagged with double-DIG. After counterstained with AP substrate NBT-BCIP (Roche) and Nuclear Fast Red™ (Vector Labs, Burlingame, CA), the *SNORD3A* and miR-185-5p transcript levels was analyzed using microscope.

Immunohistochemistry (IHC)

Immunohistochemistry staining of paraffin-embedded sections was performed according to the manufacturer instructions. Briefly, each slide was deparaffinized in xylene and rehydrated in a graded series of ethanol. After the antigen retrieval and being blocked with 5% bovine serum albumin, next, tissue sections were incubated with UMPS (Abcam, USA) or Meis1 (Abcam, USA) and then visualized by standard avidin–biotinylated peroxidase complex method. Hematoxylin was used for counterstaining and morphologic images were observed with a light microscope (Leica). Two different pathologists evaluated the immunohistological samples.
