# Supplementary figures and images for: LncRNA SNORD3A specifically sensitizes breast cancer cells to 5-FU by sponging miR-185-5p to enhance UMPS expression
Source: Cell Death Dis. 2020 May 7;11(5):329. doi: 10.1038/s41419-020-2557-2 (PMC7205983; doi:10.1038/s41419-020-2557-2)

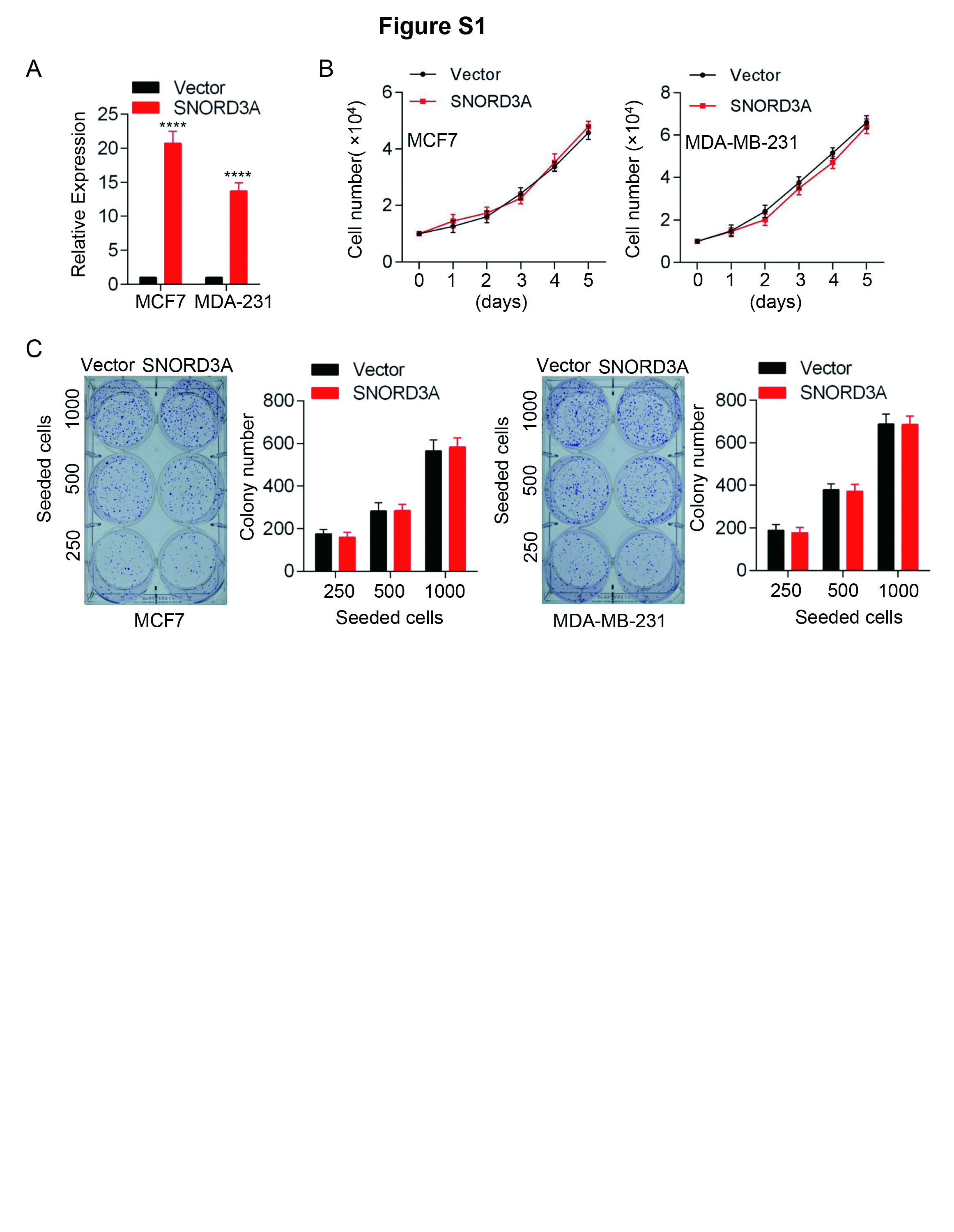

Supplement: Supplementary file 2 — Figure S1 [file 41419_2020_2557_MOESM2_ESM.tif]

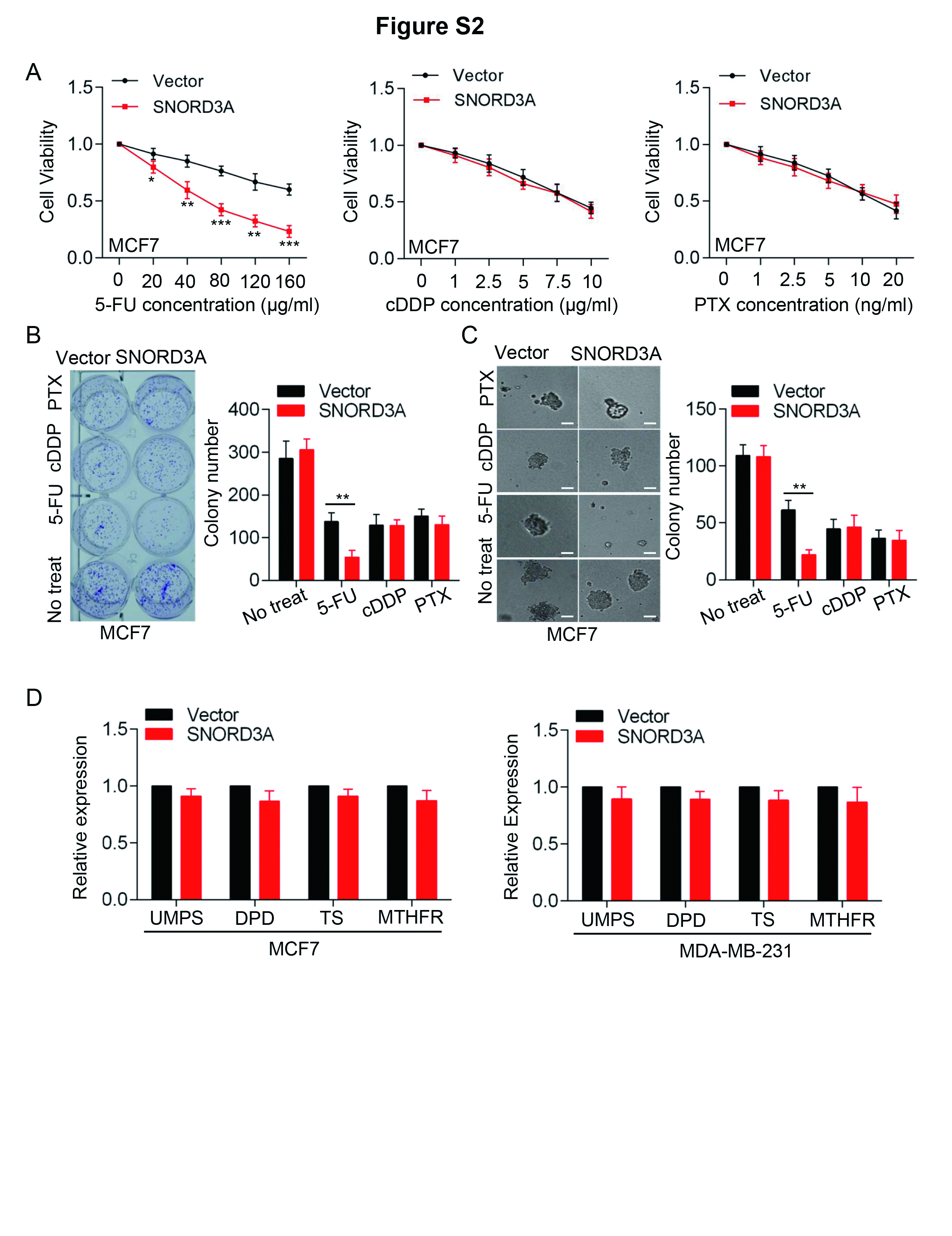

Supplement: Supplementary file 3 — Figure S2 [file 41419_2020_2557_MOESM3_ESM.tif]

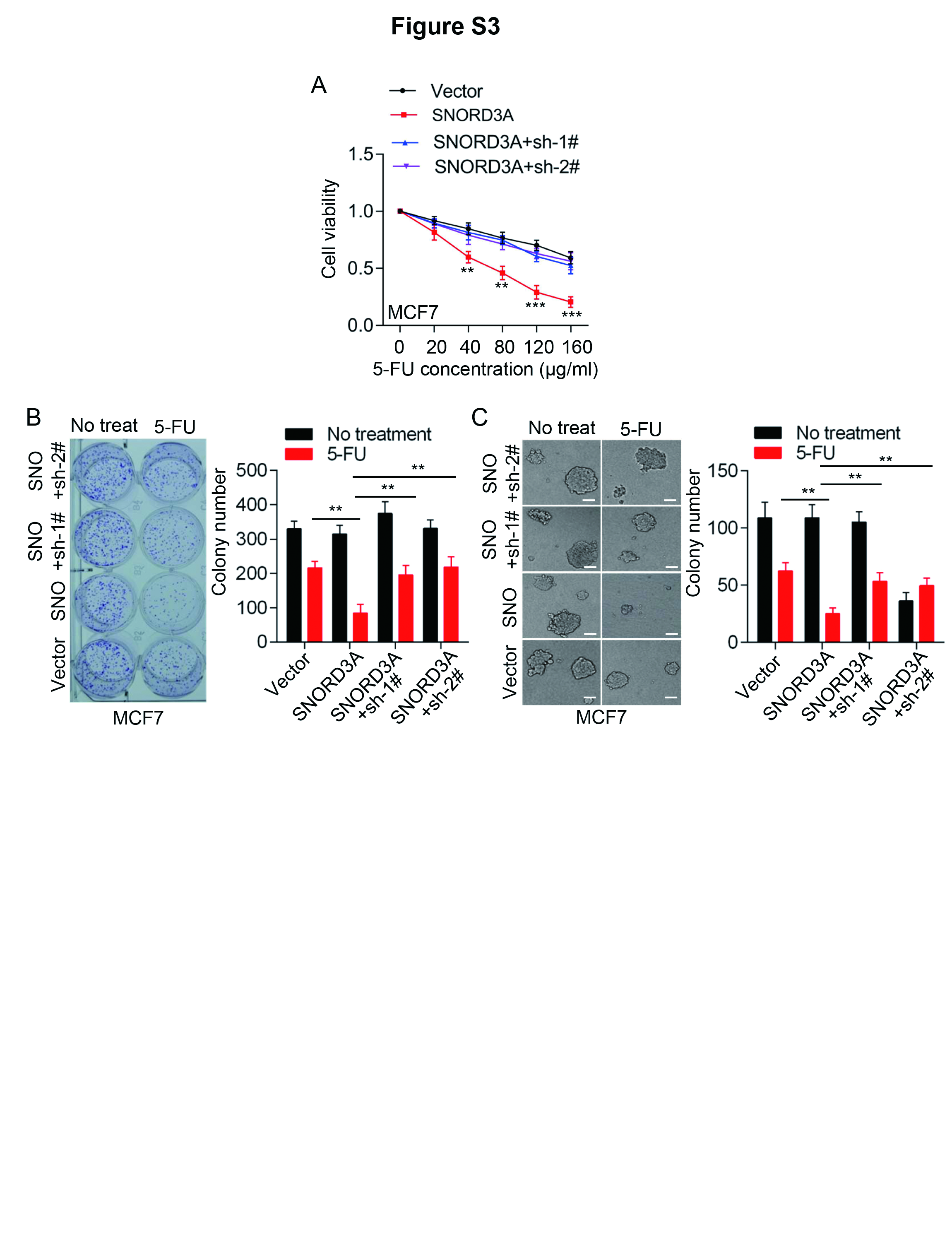

Supplement: Supplementary file 4 — Figure S3 [file 41419_2020_2557_MOESM4_ESM.tif]

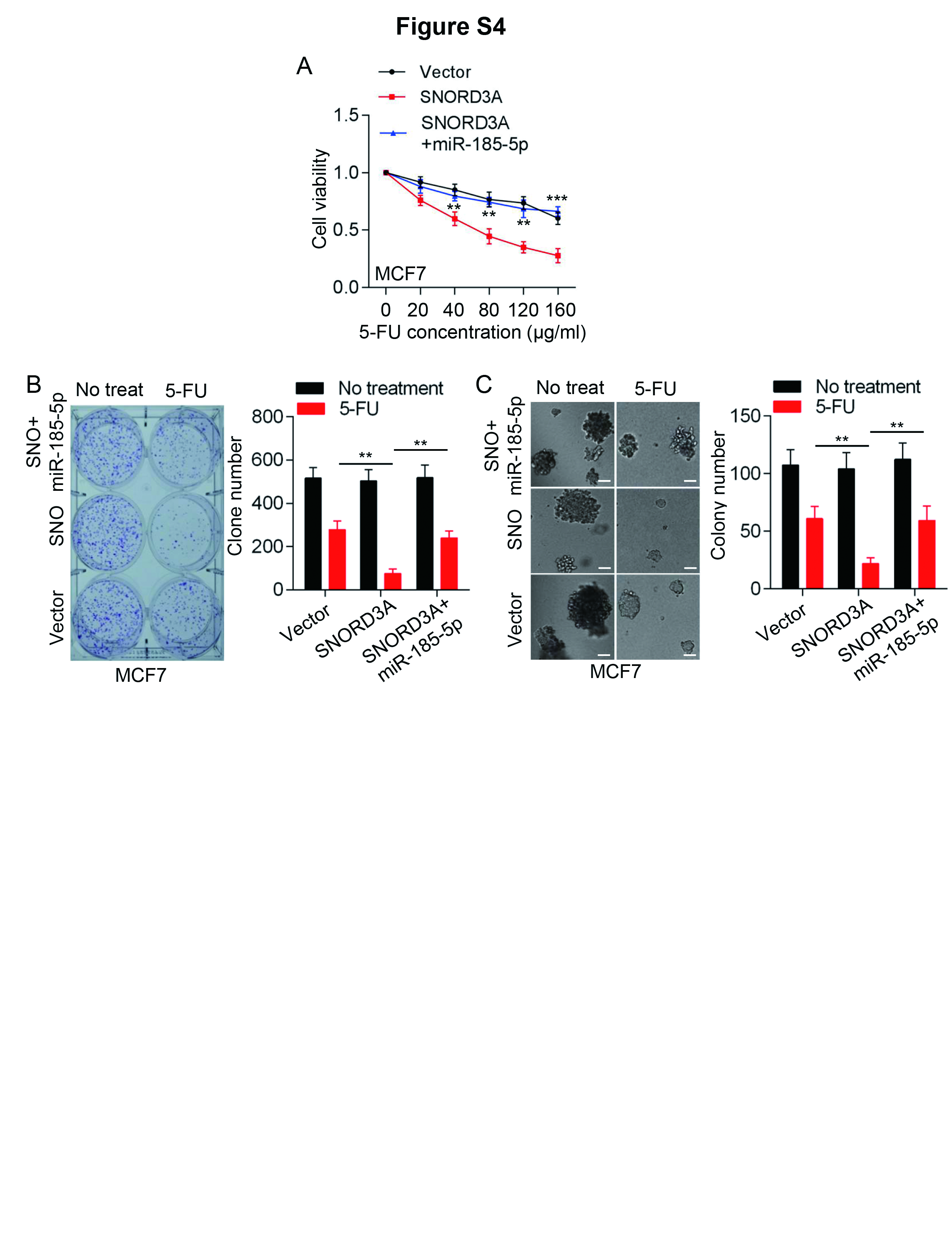

Supplement: Supplementary file 5 — Figure S4 [file 41419_2020_2557_MOESM5_ESM.tif]
